# Aspergillus fumigatus G-Protein Coupled Receptors GprM and GprJ Are Important for the Regulation of the Cell Wall Integrity Pathway, Secondary Metabolite Production, and Virulence

**DOI:** 10.1128/mBio.02458-20

**Published:** 2020-10-13

**Authors:** Aílton Pereira da Costa Filho, Guilherme Thomaz Pereira Brancini, Patrícia Alves de Castro, Clara Valero, Jaire Alves Ferreira Filho, Lilian Pereira Silva, Marina Campos Rocha, Iran Malavazi, João Guilherme de Moraes Pontes, Taícia Fill, Roberto Nascimento Silva, Fausto Almeida, Jacob L. Steenwyk, Antonis Rokas, Thaila F. dos Reis, Laure N. A. Ries, Gustavo H. Goldman

**Affiliations:** a Faculdade de Ciências Farmacêuticas de Ribeirão Preto, Universidade de São Paulo, Ribeirão Preto, Brazil; b Departamento de Genética e Evolução, Centro de Ciências Biológicas e da Saúde, Universidade Federal de São Carlos, São Carlos, Brazil; c Instituto de Química, Universidade de Campinas, Campinas, São Paulo, Brazil; d Faculdade de Medicina de Ribeirão Preto, Universidade de São Paulo, Ribeirão Preto, Brazil; e Department of Biological Sciences, Vanderbilt University, Nashville, Tennessee, USA; Duke University Medical Center

**Keywords:** *Aspergillus fumigatus*, G-protein coupled receptor, cell wall, secondary metabolites, virulence

## Abstract

G-protein coupled receptors (GPCRs) are extracellular signaling receptors that sense environmental cues. Fungi sense their environment primarily through GPCR-mediated signaling pathways, which, in turn, regulate fungal development, metabolism, virulence, and mycotoxin biosynthesis. Aspergillus fumigatus is an important human pathogen that causes aspergillosis, a heterogeneous group of diseases that present a wide range of clinical manifestations. Here, we investigate in detail the role of the GPCRs GprM and GprJ in growth and gene expression. GprM and GprJ are important for melanin production and the regulation of the cell wall integrity (CWI) pathway. Overexpression of *gprM* and *gprJ* causes a 20 and 50% reduction in growth rate compared to the wild-type (WT) strain and increases sensitivity to cell wall-damaging agents. Phosphorylation of the CWI protein kinase MpkA is increased in the Δ*gprM* and Δ*gprJ* strains and decreased in the overexpression mutants compared to the WT strain. Furthermore, differences in cell wall polysaccharide concentrations and organization were observed in these strains. Transcriptome sequencing suggests that GprM and GprJ negatively regulate genes encoding secondary metabolites (SMs). Mass spectrometry analysis confirmed that the production of fumagillin, pyripyropene, fumigaclavine C, fumiquinazoline, and fumitremorgin is reduced in the Δ*gprM* and Δ*gprJ* strains, at least partially through the activation of MpkA. Overexpression of *grpM* also resulted in the regulation of many transcription factors, with AsgA predicted to function downstream of GprM and MpkA signaling. Finally, we show that the Δ*gprM* and Δ*gprJ* mutants are reduced in virulence in the Galleria mellonella insect model of invasive aspergillosis.

## Abstract (precis)

A. fumigatus is the main etiological agent of invasive pulmonary aspergillosis, a life-threatening fungal disease that occurs in severely immunocompromised humans. Withstanding the host environment is essential for A. fumigatus virulence, and sensing of extracellular cues occurs primarily through G-protein coupled receptors (GPCRs) that activate signal transduction pathways, which, in turn, regulate fungal development, metabolism, virulence, and mycotoxin biosynthesis. The A. fumigatus genome encodes 15 putative classical GPCRs, with only three having been functionally characterized to date. In this work, we show that the two GPCRs GprM and GprJ regulate the phosphorylation of the mitogen-activated protein kinase MpkA and thus control the regulation of the cell wall integrity pathway. GprM and GprJ are also involved in the regulation of the production of the secondary metabolites fumagillin, pyripyropene, fumigaclavine C, fumiquinazoline, melanin, and fumitremorgin, and this regulation partially occurs through the activation of MpkA. Furthermore, GprM and GprJ are important for virulence in the insect model Galleria mellonella. This work therefore functionally characterizes two GPCRs and shows how they regulate several intracellular pathways that have been shown to be crucial for A. fumigatus virulence.

## INTRODUCTION

Aspergillus fumigatus is an important human pathogen that causes aspergillosis, a heterogeneous group of diseases that present a wide range of clinical manifestations, including chronic pulmonary aspergillosis (CPA), allergic bronchopulmonary aspergillosis (ABPA), and invasive pulmonary aspergillosis (IPA) (1). IPA is the most serious pathology in terms of patient outcome and treatment. It primarily affects those who are severely immunocompromised, such as patients with cancer, hematological neoplasms, or those undergoing chemotherapy (1, 2), and is accompanied by a mortality rate ranging between 50% and 100% (3).

In eukaryotes, heterotrimeric G-protein-mediated signaling pathways play a pivotal role in transmembrane signaling. G-protein coupled receptors (GPCRs) are extracellular signaling receptors that sense environmental cues which initiate intracellular G-protein signaling to coordinate a biological response. GPCRs are generally characterized by the presence of seven transmembrane (TM) domains and their association with intracellular G-proteins. The binding of an extracellular ligand to the receptor initiates intracellular signaling by stimulating the associated heterotrimeric G-proteins to exchange GDP for GTP, causing them to dissociate into the Gα subunit and a Gβ/Gγ dimer, which each have their own function in the activation or inactivation of specific pathways. G-protein functions are transient and are regulated by repressors of G-protein signaling (RGS), which promote G-protein reassociation, RGS ubiquitination, and other feedback mechanisms (4).

Fungi sense their environment primarily through GPCR-mediated signaling pathways, which, in turn, regulate fungal development, metabolism, virulence, and mycotoxin biosynthesis. Fungal GPCRs are able to detect hormones, proteins, nutrients, ions, hydrophobic surfaces, and light (5). Interestingly, fungal GPCRs do not belong to any of the mammalian receptor classes, making them specific targets for controlling fungal disease (6). There are a lot of differences in the abundance and diversity of these receptors in fungi and the potential ligands they detect. In the subkingdom of *Dikarya*, the subphylum *Pezizomycotina* contains a higher number and diversity of classical and nonclassical GPCRs than *Saccharomycotina* and *Basidiomycota* (for a review, see reference 7). Among the *Aspergillus* spp. belonging to subphylum *Pezizomycotina*, such as A. nidulans, A. flavus, and A. fumigatus, the number of classical GPCRs in each species is highly conserved (7).

In A. fumigatus, 161 proteins were found to encode seven predicted transmembrane domains (TMDs) (8); however, 15 putative classical GPCRs have been identified in this pathogenic fungus (8, 9). Deletion of *gprC* and *gprD* resulted in strains with severe growth defects, as hyphal extension was reduced, germination was retarded, and hyphae showed elevated levels of branching (10). Furthermore, the Δ*gprC* and Δ*gprD* strains were more sensitive to oxidative stress and more tolerant to cyclosporine (an inhibitor of the protein phosphatase calcineurin) and displayed attenuation of virulence in a murine infection model (10). Deletion of *gprK* resulted in a strain with reduced conidiation and an increased germination rate, and GprK was shown to control fungal development through the cAMP-protein kinase A (PKA) pathway to regulate the expression of developmental genes (11). GprK was also proposed to be involved in sensing pentose sugars and in the control of the oxidative stress response through regulating the expression of catalase- and superoxide dismutase-encoding genes via the mitogen-activated protein kinase (MAPK) SakA signaling pathway (11). In addition, GprK is important for the production of the secondary metabolite (SM) gliotoxin, although this GPCR was dispensable for virulence in the Galleria mellonella insect model of invasive aspergillosis (11).

Recently, we showed that the GPCR GprM is a negative regulator of melanin production, as its deletion results in an increase in the melanin dihydroxynaphthalene (DHN) (12). Melanins are a class of dark-brown pigments often associated with the cell wall. Their main role is to protect the organisms from exogenous stressors, thereby contributing to the first line of defense against external hazards (13, 14). A. fumigatus produces two types of melanins: pyomelanin, which is derived from the catabolism of tyrosine via the intermediate homogentisate, and DHN-melanin, which is produced as a polyketide derivative and is responsible for the gray-green color of the spores (13). Similar phenotypes were observed in strains deleted for the Gα protein GpaA and the MAPK MpkB (12), suggesting that these three proteins function in the same pathway. Split ubiquitin-based membrane yeast two-hybrid and coimmunoprecipitation assays confirmed that GpaA and GprM interact, suggesting their role in the MpkB signaling cascade (12).

Apart from the aforementioned studies, other A. fumigatus GPCRs have not been functionally characterized, and we currently have very limited understanding of how this pathogenic fungus can sense host cues. In this work, we further investigated the role of GprM and also of GprJ in fungal growth, gene expression, cell wall integrity, SM production, and virulence. Both GPCRs are important for the regulation of the cell wall integrity (CWI) pathway and the production of several SMs in addition to contributing to A. fumigatus virulence.

## RESULTS

### The GPCRs GprM and GprJ are important for melanin production and the regulation of the CWI pathway.

**FIG 1:**
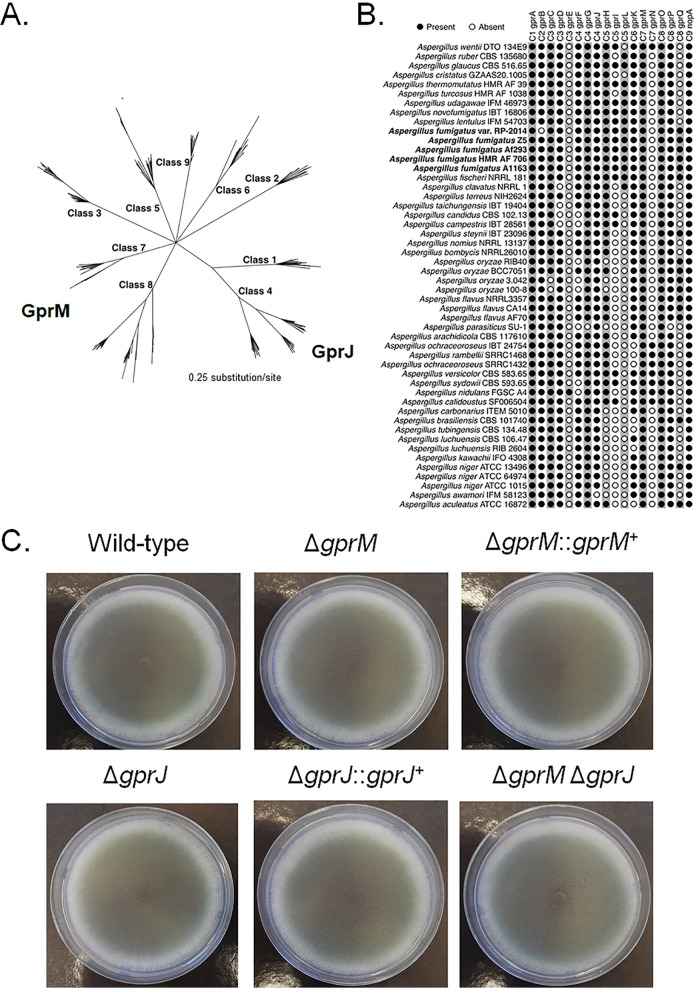
A. fumigatus GprM and GprJ homologues are present in different *Aspergillus* spp. (A) Phylogenetic relationship among the nine classes of GPCRs *present in aspergilli*. GprM is a class 7 GPCR, whereas GprJ is a class 4 GPCR. (B) Distribution of 18 A. fumigatus GPCR homologues in *Aspergillus* species. A. fumigatus strains are highlighted in bold. The presence of a GPCR is marked by a black circle, whereas the absence is indicated by a white circle. (C) The knockout of *gprM* and *gprJ* in single- and double-deletion strains does not affect fungal growth on glucose minimal medium. Strains were grown from 10^5^ spores for 5 days at 37°C.

**FIG 2:**
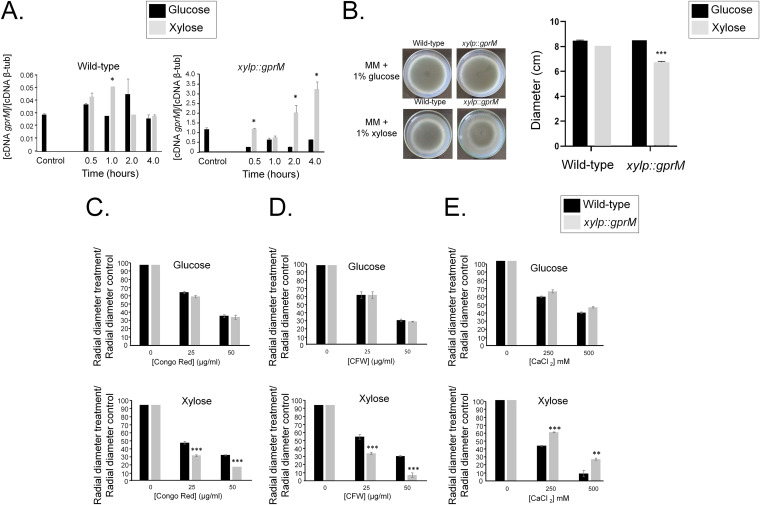
Overexpression of *gprM* causes decreased growth fitness and increased sensitivity to cell wall-damaging agents. (A) Overexpression of *gprM* was confirmed by reverse transcriptase quantitative PCR (RT-qPCR). Strains were grown for 24 h at 37°C in glucose minimal medium (GMM) (control) and transferred to xylose minimal medium (XMM) for 0.5, 1, 2, and 4 h before RNA was extracted and reverse transcribed to cDNA. Copy numbers of *gprM* were normalized by β-tubulin (AspGD database no. Afu1g10910). (B) Overexpression of *gprM* affects growth. Strains were grown from 10^5^ spores for 5 days at 37°C on GMM or XMM before radial diameter was measured. Pictures on the left are indicative for measurements of the graph shown on the right. (C to E) Overexpression of *gprM* increases sensitivity to cell wall-damaging agents. Strains were grown from 10^5^ spores for 5 days at 37°C on GMM or XMM supplemented with increasing concentrations of Congo red (C), calcofluor white (CFW) (D), and calcium chloride (CaCl2) (E). Growth was normalized by growth in the control, drug-free condition. Standard deviations present the average of three independent biological repetitions. *, *P* < 0.05, **, *P* < 0.005, and ***, *P* < 0.0005 in a one-way ANOVA with Tukey’s test for *post hoc* analysis compared to the wild-type strain.

**FIG 3:**
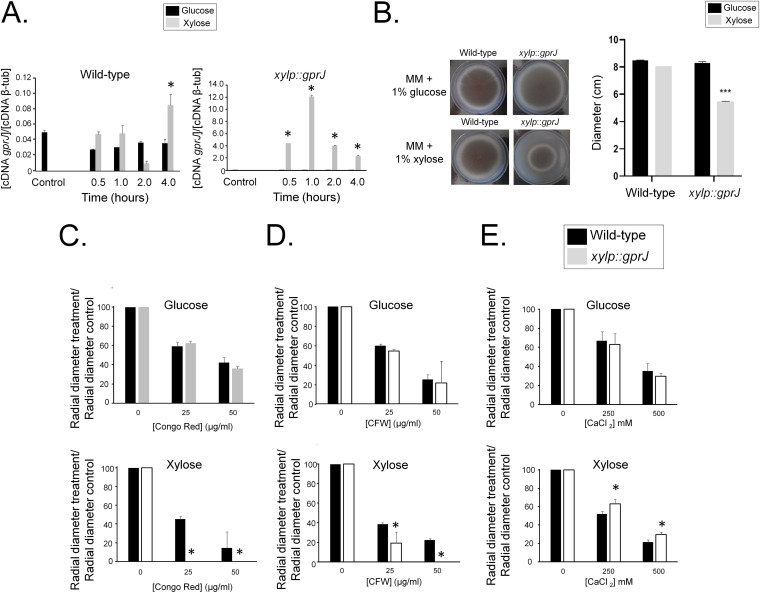
Overexpression of *gprJ* causes decreased growth fitness and increased sensitivity to cell wall-damaging agents. (A) Overexpression of *gprJ* was confirmed by RT-qPCR. Strains were grown for 24 h at 37°C in glucose minimal medium (GMM) (control) and transferred to xylose minimal medium (XMM) for 0.5, 1, 2, and 4 h before RNA was extracted and reverse transcribed to cDNA. Copy numbers of *gprM* were normalized by β-tubulin (AspGD database no. Afu1g10910). (B) Overexpression of *gprJ* affects growth. Strains were grown from 10^5^ spores for 5 days at 37°C on GMM or XMM before radial diameter was measured. Pictures on the left are indicative of measurements of the graph shown on the right. (C to E) Overexpression of *gprJ* increases sensitivity to cell wall-damaging agents. Strains were grown from 10^5^ spores for 5 days at 37°C on GMM or XMM supplemented with increasing concentrations of Congo red (C), calcofluor white (CFW) (D), and calcium chloride (CaCl_2_) (E). Growth was normalized by growth in the control, drug-free condition. Standard deviations present the average of three independent biological repetitions. *, *P* < 0.05, **, *P* < 0.005, and ***, *P* < 0.0005 in a one-way ANOVA with Tukey’s test for *post hoc* analysis compared to the wild-type strain.

Previously, we have shown that the MAPK MpkB is important for conidiation and that its deletion increases dihydroxynaphthalene (DHN)-melanin production, as observed by a dark coloring of liquid culture supernatants (12). Culture supernatants of strains deleted for the Gα protein GpaA and for the G-protein coupled receptor GprM also turned dark (12). To identify additional pathways involved in DHN-melanin regulation, we exploited the dark-color production in the supernatant as a readout system. Using the same methodology, we screened 12 A. fumigatus GPCR deletion strains (Table S1 at https://doi.org/10.6084/m9.figshare.12869864) in order to determine whether additional GPCRs are involved in melanin production. Dark-colored supernatants were observed for the Δ*gprM* and Δ*gprJ* strains compared to the wild-type (WT) strain but not for the Δ*gprM* Δ*gprJ* strain (Fig. S1 at https://doi.org/10.6084/m9.figshare.12869864). Complementation of Δ*gprJ* and simultaneous deletion of the *pksP* gene, encoding the polyketide synthase PksP required for DHN-melanin biosynthesis, confirm that the change in culture medium was linked to DHN-melanin (Fig. S1 at https://doi.org/10.6084/m9.figshare.12869864). *In silico* homology and similarity analyses group GprM and GprJ as class 7 (orthologues of MG00532 from Magnaporthe oryzae with weak similarity to the rat growth hormone-releasing factor) and class 4 (nitrogen) GPCRs, respectively (7) (Fig. 1A). GprM and GprJ are present in 45 *Aspergillus* species (except A. awamori for GprJ), and both GPCRs are present in five different A. fumigatus strains (Fig. 1B). In addition to the *gprM* and *gprJ* single- and double-deletion strains, we constructed the corresponding *gprM* and *gprJ* overexpression strains (Table S1 at https://doi.org/10.6084/m9.figshare.12869864). These deletion strains have comparable growth and conidiation to the wild-type strain on solid (Fig. 1C) and in liquid (Fig. S2A at https://doi.org/10.6084/m9.figshare.12869864) minimal medium (MM). The overexpression strains (Table S1 at https://doi.org/10.6084/m9.figshare.12869864) were constructed by replacing the *gprM* and *gprJ* endogenous promoter by the inducible *xyl* promoter from Penicillium chrysogenum, which is induced by xylose and repressed by glucose (15). Overexpression was confirmed by reverse transcription-quantitative PCR (qRT-PCR) (Fig. 2A and 3A). Induction of *gprM* was increased 66- and 106-fold in the *xylP::gprM* strain when grown for 2 and 4 h in xylose, respectively, in comparison to the WT strain (Fig. 2A). Induction of *gprJ* was increased 100- and 260-fold in the *xylP::gprJ* strain when grown for 30 min and 1 h in xylose-rich medium in comparison to the WT strain (Fig. 3A).

We then grew all strains in glucose minimal medium (GMM) supplemented with increasing concentrations of cell wall-perturbing agents. All growth was normalized in comparison to the drug-free control condition. The Δ*gprM*, Δ*gprJ*, and Δ*gprM* Δ*gprJ* strains were not sensitive to these compounds. In contrast, the *gprM* overexpression strain has a 20% reduction in growth compared to the WT strain when grown on xylose minimal medium (XMM) (Fig. 2B). Furthermore, *gprM* overexpression causes a significant reduction in growth in the presence of Congo red (CR) and calcofluor white (CFW) (Fig. 2C and D). The *xylP::gprM* strain also presented increased growth in the presence of CaCl_2_ (Fig. 2E). Similar to the *xylP::gprM* strain, overexpression of *gprJ* resulted in 50% reduced growth compared to the wild-type strain in the presence of XMM (Fig. 3B) and in significantly reduced growth in the presence of CR and CFW (Fig. 3C and D) and increased growth in the presence of CaCl_2_ (Fig. 3E).

**FIG 4:**
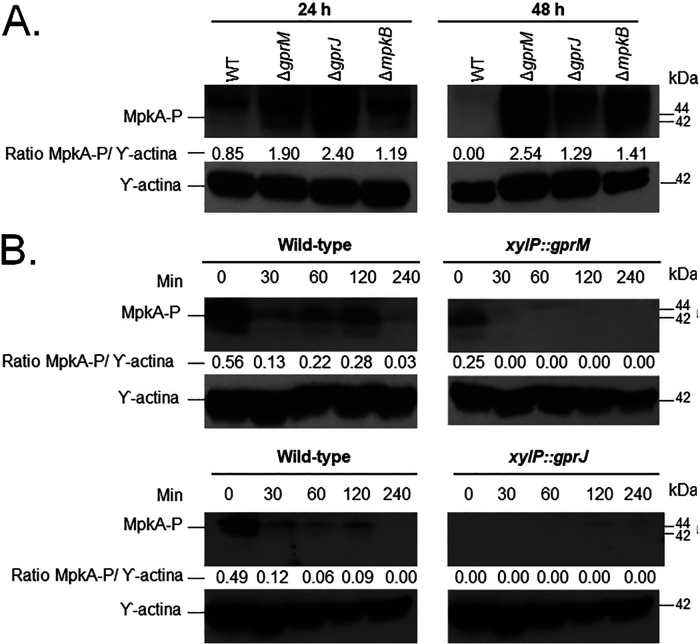
GprM and GprJ affect MpkA phosphorylation. (A) Deletion strains were grown for 24 or 48 h in glucose minimal medium (GMM). (B) Overexpression strains were grown in MM before mycelia were transferred to xylose minimal medium for 30, 60, 120, and 240. Total cellular proteins were extracted and quantified, and Western blotting was run against phosphorylated MpkA (MpkA-P) using the anti-44/42 MpkA antibody. MpkA phosphorylation signals were normalized by cellular γ-actin using the anti-γ-actin antibody. Signal intensities were quantified using the ImageJ software, and ratios of MpkA-P to γ-actin were calculated.

Due to the increased sensitivity of the *gprM* and *gprJ* overexpression strains to cell wall-perturbing compounds, we further investigated the role of GprM and GprJ in cell wall maintenance. A. fumigatus MpkA is the main MAPK responsible for the CWI pathway and cell wall remodeling with MpkA phosphorylation signaling CWI pathway activation (16). We investigated the impact of the deletion and overexpression of *gprM* and *gprJ* on MpkA phosphorylation (Fig. 4). We used the Δ*mpkB* strain as a control because it was shown to have increased MpkA phosphorylation after 48 h growth in GMM compared to the WT strain (12) (Fig. 4A). To normalize the observed strain-specific phosphorylated MpkA levels, we used anti-β-actin as the antibody that detects total cellular protein since the anti-p42-44 antibody to detect total MpkA does not function for A. fumigatus cellular protein extracts. MpkA phosphorylation was increased after 24 and 48 h growth in GMM in the Δ*gprM* and Δ*gprJ* strains compared to the WT strain (Fig. 4A). In contrast, overexpression of *gprM* and *gprJ* caused a decrease in MpkA phosphorylation compared to the WT strain (Fig. 4B). The Δ*gprM* Δ*gprJ* strain had reduced MpkA phosphorylation compared to the WT strain (data not shown), suggesting that they genetically interact in the regulation of the CWI pathway.

**FIG 5:**
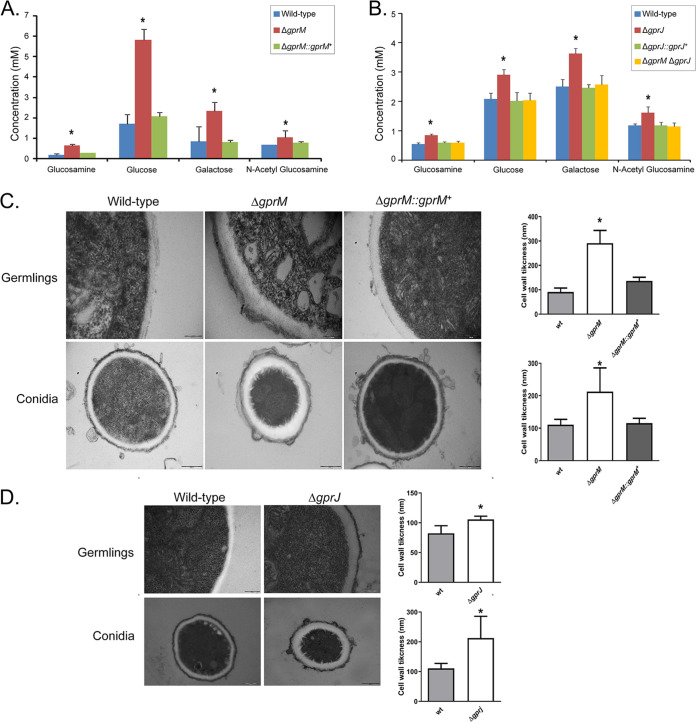
GprM and GprJ affect the cell wall organization. (A and B) Single deletion of *gprM* and *gprJ* results in increased concentrations of cell wall sugars. Strains were grown for 24 h in glucose minimal medium (GMM) before cell wall-soluble fractions were prepared and high-performance liquid chromatography (HPLC) was performed. Standard deviations present the average of six biological repetitions *, *P* < 0.05 in a one-way ANOVA test in comparison to the wild-type strain. (C and D) Transmission electron microscopy of hyphal germlings and conida of the Δ*gprM* (C) and Δ*gprJ* (D) strains when grown for 24 h in GMM. Shown are representative pictures of hyphal and conidia cell wall sections as well as graphs showing the average cell wall thickness (nm) of 100 sections of different hyphal germlings or conidia (average of 4 sections per germling). Standard deviations present the average of 100 measurements. *, *P* < 0.00001 in a one-tailed, paired *t* test in comparison to the wild-type (WT) strain.

To further investigate the role of GprM and GprJ in cell wall maintenance, we measured concentrations of cell wall polysaccharides and cell wall thickness in the respective deletion strains. Deletion of *gprM* and *gprJ* resulted in increased concentrations of cell wall glucosamine, glucose, galactose and *N*-acetylglucosamine, whereas the simultaneous deletion of *gprM* and *gprJ* restored cell wall sugar concentrations back to levels observed in the WT and complemented strains (Fig. 5A and B). Transmission electron microscopy analysis of Δ*gprM* and Δ*gprJ* hyphal germlings and conidia showed that their cell walls were significantly thicker than the cell walls of the WT and complemented strains (Fig. 5C and D; Table S2 at https://doi.org/10.6084/m9.figshare.12869864). Together, these results suggest that GprM and GprJ regulate cell wall maintenance, likely through the MpkA CWI pathway. However, the simultaneous absence of both GPCRs activates an unknown compensatory signaling mechanism that maintains normal cell wall organization.

### GprM affects protein kinase A activity.

**FIG 6:**
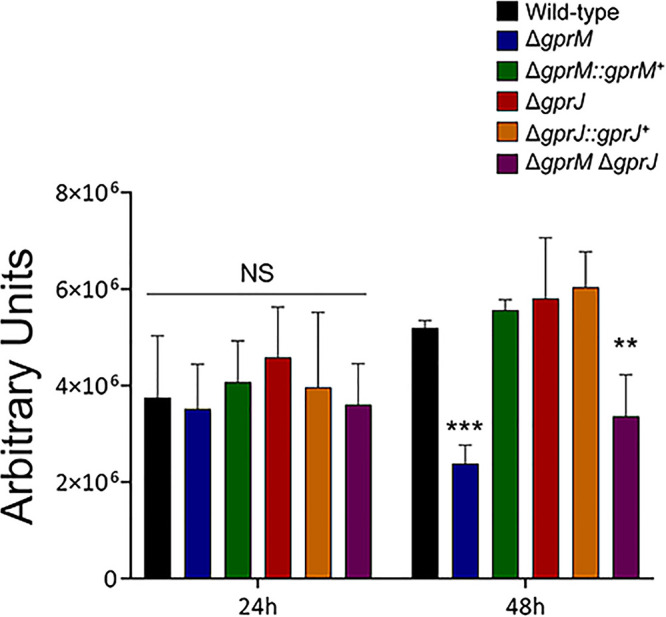
Protein kinase A (PKA) activity is reduced in the Δ*gprM* and Δ*gprM* Δ*gprJ* mutants. Strains were grown for 24 or 48 h in glucose minimal medium before total cellular proteins were extracted and PKA activity was measured. Standard deviations present the average of three biological replicates. **, *P* < 0.01 and ***, *P* < 0.001 (NS, nonsignificant) in a two-way ANOVA followed by Bonferroni posttests.

The cAMP-PKA pathway is activated in response to GPCR heterotrimeric G-protein signaling and regulates a variety of cellular processes, including fungal development and carbon source (e.g., glucose) utilization in *Aspergillus* species (7, 17). Furthermore, the cAMP-PKA pathway is also important for sugar metabolism, carbohydrates that are building blocks for cell wall polysaccharide precursors in A. fumigatus (18). We investigated whether cAMP-PKA signaling is activated by GprM and GprJ in the presence of glucose. PKA activity in the single and double mutants is comparable to the WT strain after 24 h growth in GMM, but Δ*gprM* and Δ*gprM* Δ*gprJ* have significantly reduced PKA activity after 48 h growth in GMM (Fig. 6). Reduction of PKA activity in the Δ*gprM* Δ*gprJ* had no additive interaction, as PKA activity in the double mutant was mostly reminiscent of Δ*gprM*. Growth in the presence of glucose and glucose transport were not significantly different in the single and double *gprM* and *gprJ* deletion strains than from the WT strain (Fig. S2 at https://doi.org/10.6084/m9.figshare.12869864). Together, these results suggest that GprM, but not GprJ, activates the cAMP-PKA signaling pathway and that, in this case, the cAMP-PKA pathway is required for regulation of cellular processes that are not related to glucose metabolism.

### Transcriptional profiling of the *gprM* and *gprJ* loss of function and overexpression strains.

To determine which genes are under the regulatory control of GprM and GprJ, we performed transcriptome sequencing of the single deletion strains in GMM and of the overexpression strains in the presence of GMM and XMM. Differentially expressed genes (DEGs) were defined as those with a minimum log_2_ fold change (log_2_FC) of 1 (log_2_FC ≥ 1.0 and ≤ −1.0; *P* < 0.05; FDR [false discovery rate], 0.05) when comparing the deletion or overexpression strains to the WT strain.

**FIG 7:**
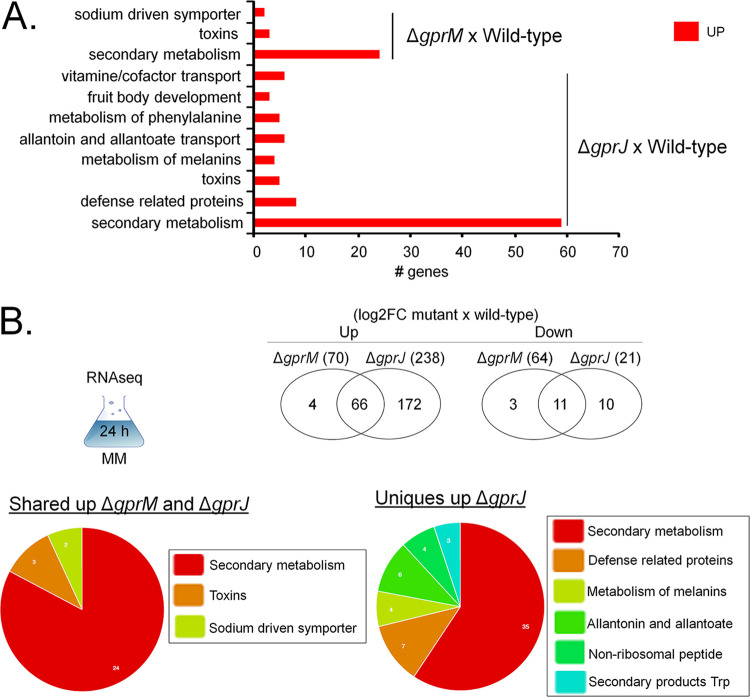
Functional characterization (FunCat) of significantly differently expressed genes (DEGs) identified by RNA-seq in the Δ*gprM* and Δ*gprJ* strains. (A) FunCat analysis of DEGs upregulated in the Δ*gprM* and Δ*gprJ* strains in comparison to the wild-type (WT) strain when grown for 24 h in glucose minimal medium (GMM). (B) Venn diagrams showing the number of up- or downregulated DEGs specific to each deletion strain and shared among the Δ*gprM* and Δ*gprJ* strains in comparison to the WT strain. In parentheses is the total number of DEGs identified for each strain (log_2_FC). A diagram depicting the growth condition used for RNA-seq is also shown. In addition, FunCat analyses for upregulated DEGs shared among the two deletion strains and for the Δ*gprJ* strain in comparison to the WT are shown as pie charts.

The WT, Δ*gprM*, and Δ*gprJ* transcriptional response was assessed after 24 h growth in GMM. In the Δ*gprM* and Δ*gprJ* strains, 70 and 238 genes were upregulated, respectively, whereas 64 and 21 genes were downregulated, respectively (Fig. 7A). Of these, 66 and 11 genes were up- and downregulated, respectively, in both deletion strains (Fig. 7A). Functional characterization (FunCat) (https://elbe.hki-jena.de/fungifun/fungifun.php) enrichment analyses for the Δ*gprM* and Δ*gprJ* strains demonstrated a transcriptional upregulation of genes coding for proteins involved in secondary metabolism, toxins, and metabolism of melanins (Fig. 7B). FunCat analysis did not show any significant enrichment for the downregulated genes, probably due to the low number of DEGs. We then focused on the FunCat enrichment analysis of DEGs common to both strains and specific for each deletion strain. Most of the upregulated DEGs shared between the mutants encode proteins involved in secondary metabolism (Fig. 7C). The 172 DEGs specifically upregulated in the Δ*gprJ* strains encoded proteins involved in secondary metabolism, metabolism of melanins, and nonribosomal peptides (Fig. 7C).

**FIG 8:**
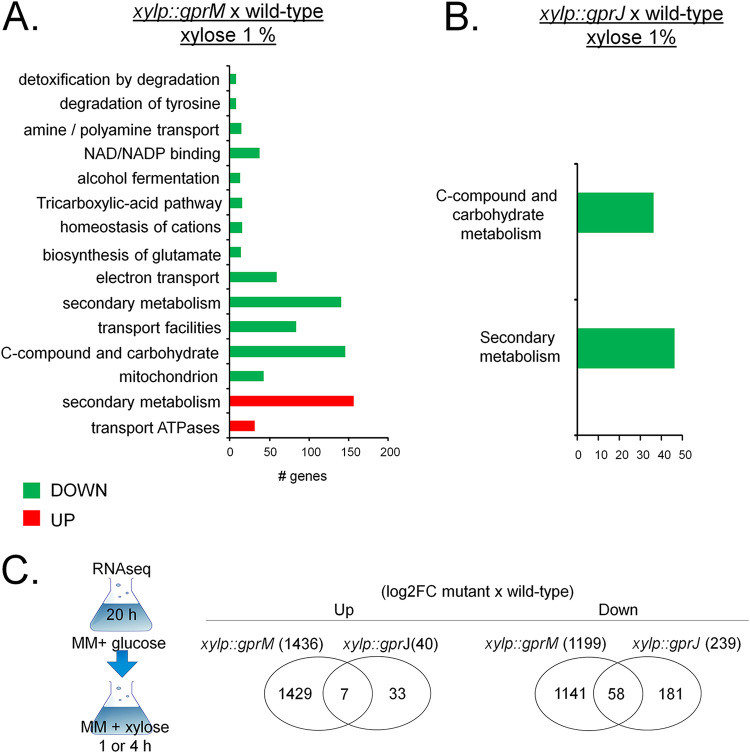
Functional characterization (FunCat) of significantly differently expressed genes (DEGs) identified by RNA-seq in the *gprM*- and *gprJ*-overexpressing strains. (A) FunCat analysis of DEGs up- and downregulated in the *gprM*-overexpressing strain in comparison to the wild-type (WT) strain when grown for 4 h in xylose minimal medium (XMM) after transfer from 24 h growth in glucose minimal medium (GMM). (B) FunCat analysis of DEGs downregulated in the *gprJ*-overexpressing strain in comparison to the WT strain when grown for 1 h in XMM after transfer from 24 h growth in GMM. (C) Venn diagrams showing the number of up- or downregulated DEGs specific to each deletion strain and shared among the Δ*gprM* and Δ*gprJ* strains in comparison to the WT strain (log_2_FC). In parentheses is the total number of DEGs identified for each strain. A diagram depicting the growth condition used for RNA-seq is also shown.

Next, we analyzed the transcriptional profiles of GprM and GprJ in conditions of gene overexpression. The *xylP::gprM* and *xylp::gprJ* strains were grown for 16 h in GMM before being transferred to 1% wt/vol xylose MM for 4 h (*xylP::gprM*) and 1 h (*xylp::gprJ*) to induce gene overexpression (Table S5 and S6 at https://doi.org/10.6084/m9.figshare.12869864). In the *xylP::gprM* and *xylp::gprJ* strains, 1,436 and 40 and 1,199 and 239 genes were significantly upregulated and downregulated, respectively (Fig. 8A and Table S5 and S6 at https://doi.org/10.6084/m9.figshare.12869864). Of these, 7 and 58 genes were up- and downregulated, respectively, in both overexpression strains (Fig. 8A). FunCat enrichment analyses of the *xylP::gprM* strain showed a transcriptional upregulation of genes encoding proteins involved in secondary metabolism and transport ATPases and transcriptional downregulation of genes coding for proteins involved in electron transport, secondary metabolism, transport facilities, C-compound and carbohydrate metabolism, and mitochondrion (Fig. 8B). FunCat enrichment analysis of upregulated DEGs in the *xylp::gprJ* could not be carried out due to the low number of DEGs. FunCat analysis of downregulated DEGs in the *xylp::gprJ* strain showed enrichment of C-compound and carbohydrate metabolism and secondary metabolism enrichment (Fig. 8C). We were unable to carry out FunCat analyses for the DEGs that are under the regulatory control of both GPCRs, which was again due to the low number of DEGs.

Taken together, the data suggest that GprM and GprJ each regulate a unique set of genes and that not many genes are target to regulation by both GPCRs in the conditions tested here.

### GprM and GprJ are important for the regulation of the production of SMs.

**FIG 9:**
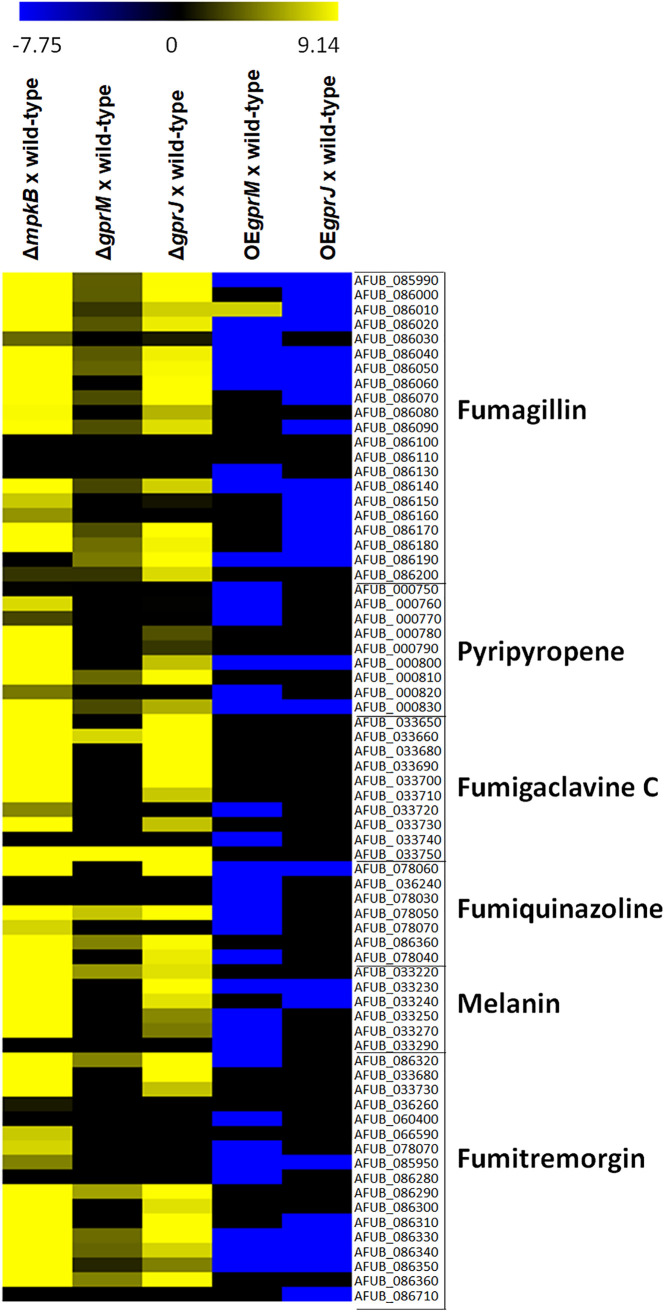
GprM and GprJ are important for the regulation of genes encoding proteins involved in secondary metabolite (SM) biosynthesis. Heat map depicting the log_2_ fold change of differentially expressed genes (DEGs), as determined by RNA-seq, and encoding enzymes required for specific SM biosynthesis. Log_2_FC values are based on comparisons between the *mpkB* (control), *gprM* and *gprJ* deletion strains, and the wild-type strain; as well as between the *gprM*- and *gprJ*-overexpressing (OE) strains and the wild-type strain. Heat map scale and gene identities are indicated. Hierarchical clustering was performed in Multiple Experiment Viewer (MeV) (http://mev.tm4.org/), using Pearson correlation with complete linkage clustering.

We then focused on genes that were differentially expressed in our transcriptome sequencing (RNA-seq) data and encoded SM biosynthesis proteins, as we hypothesized that GprM and GprJ are involved in the regulation of SM biosynthesis. We also added the transcriptional profile of Δ*mpkB* when grown for 24 h in GMM to this analysis as a control (Fig. 9; Table S7 at https://doi.org/10.6084/m9.figshare.12869864). Visual inspection of DEGs encoding SM-related proteins showed that biosynthetic gene clusters (BGC) of fumagillin, pyripyropene, fumigaclavine C, fumiquinazoline, melanin, and fumitremorgin were upregulated in the Δ*gprM*, Δ*gprJ*, and Δ*mpkB* strains and downregulated in the *xylP::gprM* and *xylp::gprJ* strains, suggesting that GprM and GprJ are important for the transcriptional regulation of SM BGCs (Fig. 9).

**FIG 10:**
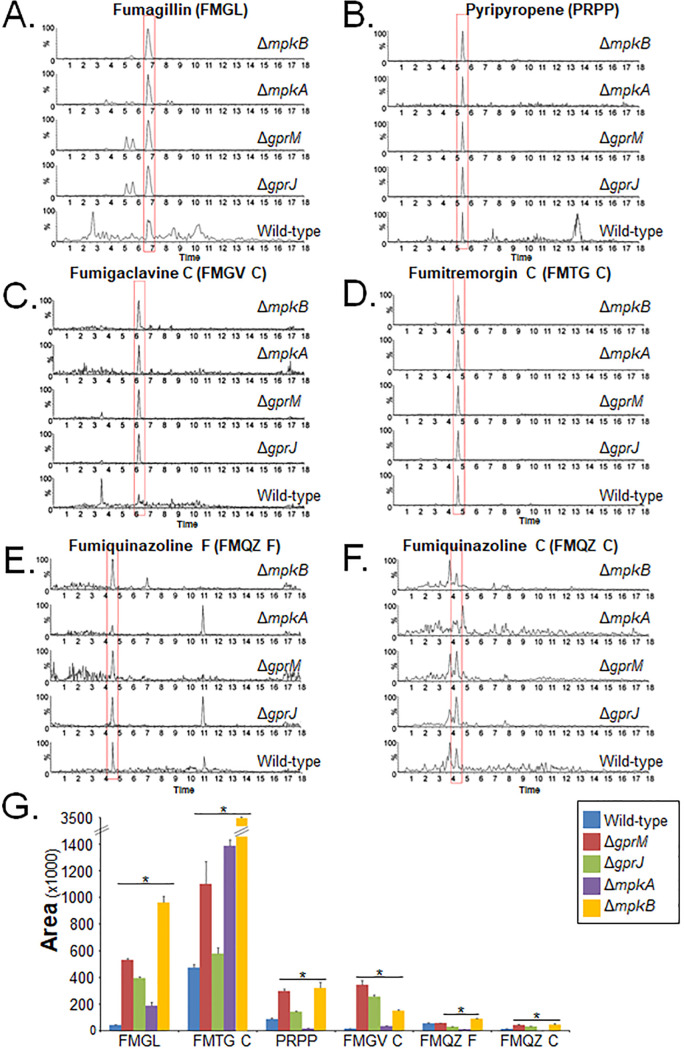
GprM and GprJ are involved in the repression of several secondary metabolites (SMs). SMs were extracted from culture supernatants of strains grown for 120 h in glucose minimal medium before mass spectrometry (MS) was performed. (A to F) MS chromatograms of fumagillin (A), pyripyropene A (B), fumigaclavine C (C), fumitremorgin C (D), fumiquinazoline F (E), and fumiquinazoline C (F) generated for different strains. (G) Quantitative comparison of the SMs identified in panels A to F between the deletion and the wild-type strains. Standard deviations represent the average of biological triplicates with *, *P* < 0.05 in a two-tailed unpaired Student's *t* test.

To confirm a role of both GPCRs in SM production, mass spectrometry (MS) analysis of supernatants of strains grown for 5 days in MM at 37°C was carried out. MS analysis detected the presence of increased levels of fumagillin, pyripyropene, fumigaclavine C, fumitremorgin C, and fumiquinazolines F and C in the Δ*gprM*, Δ*gprJ*, and Δ*mpkB* strains compared to the WT strain (Fig. 10A to G). These results are in agreement with the RNA-seq data. Since Δ*gprM*, Δ*gprJ*, and Δ*mpkB* strains have increased MpkA phosphorylation (Fig. 4), we wondered whether the production of these SMs was also altered in the Δ*mpkA* strain (Fig. 10A to G). The Δ*mpkA* strain had increased production of fumagillin and fumitremorgin in comparison to the WT strain, whereas concentrations of pyripyropene A, fumigaclavine C, and fumiquinazolines F and C were reduced in this strain (Fig. 10A to 10G).

These results indicate that GprM and GprJ negatively regulate the production of several secondary metabolites and that this regulation occurs through the CWI pathway in an SM-dependent manner.

### Screening of an A. fumigatus TF deletion library identifies a TF important for CWI.

**FIG 11:**
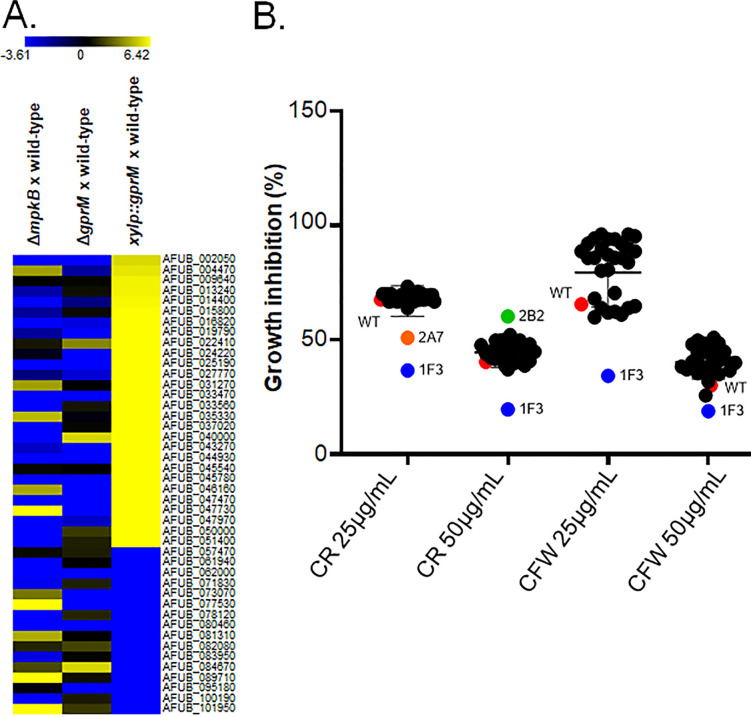

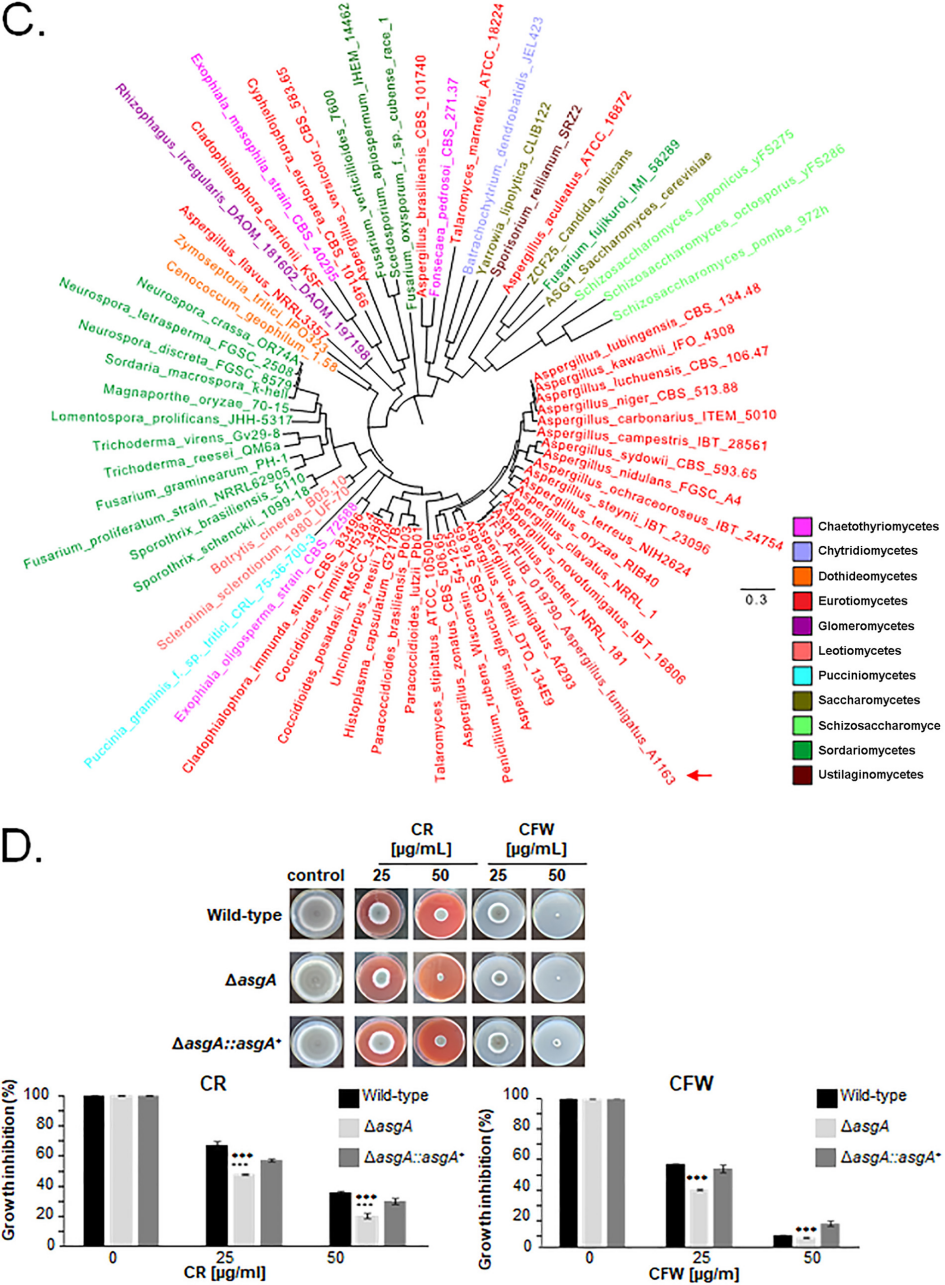
Identification of a TF involved in mediating the response to cell wall damage. (A) Heat maps depicting the log_2_ fold change of 44 differentially expressed genes (DEGs), as determined by RNA-seq, and encoding transcription factors (TFs) that were modulated in the *xylP::gprM* strain. Log_2_FC values are based on comparisons between the *mpkB* and *gprM* deletion strains and the *gprM*-overexpressing (*xylP::gprM*) strain and the wild-type strain. Heat map scale and gene identities are indicated. Hierarchical clustering was performed in MeV (http://mev.tm4.org/), using Pearson correlation with complete linkage clustering. (B) Identification of the TF AsgA as important for cell wall stress resistance. Graph showing the percentage of growth of 44 TF deletion strains (named by simple numbers and letters) in the presence of different concentrations of the cell wall-damaging agents Congo red (CR) and calcofluor white (CFW). The growth percentage was calculated by dividing colony diameter in the presence of the drug by the colony diameter in the control, drug-free condition specific to each strain. The 1F3 strain, subsequently named Δ*asgA* strain, was significantly more sensitive to the cell wall-perturbing compounds. (C) The phylogenetic distribution of AsgA across fungal classes and genomes. Sequences were aligned through ClustalW implemented in Molecular Evolutionary Genetics Analysis 7 (MEGA7) software (59). Phylogenetic analyzes were performed using the MEGA7 software, using the neighbor-joining method (60) and 1,000 bootstrap replications (61) for each analysis. The phylogenetic tree was visualized using the FigTree program (http://tree.bio.ed.ac.uk/software/figtree/). The red arrow indicates the *asgA* gene. (D) Confirmation of AsgA-mediated sensitivity to CR and CFW. Strains were grown from 10^5^ spores for 5 days at 37°C on glucose minimal medium supplemented with increasing concentrations of CR and CFW before pictures were taken (left) and radial diameter of biological triplicates was measured (right). Standard deviations represent the average of three biological replicates. ***, *P* < 0.0005 in a one-tailed, paired *t* test in comparison to the wild-type (WT) strain.

To identify effector components that act downstream of GprM and the CWI pathway, we screened our RNA-seq data for DEGs encoding transcription factors (TFs) and found 44 genes of which 28 were upregulated and 16 were downregulated in the *xylP::gprM* strain when grown for 4 h in XMM (Fig. 11A). For this analysis, we focused on GprM because this receptor was shown to be important for PKA activity (Fig. 6), a protein kinase that is involved in cell wall maintenance (18). Some of these genes were also modulated in the Δ*mpkB* and Δ*gprM* strains, but not in the Δ*gprJ* and *xylp::gprJ* strains (Fig. 11A and Table S3 to S6 and Table S8 at https://doi.org/10.6084/m9.figshare.12869864). The TF deletion strains were grown on GMM supplemented with increasing concentrations of CR and CFW before radial diameter was measured and normalized by the drug-free control condition (Fig. 11B). The 1F3 (ΔAFUB_019790) strain was significantly more sensitive to CR and CFW (Fig. 11B). The ΔAFUB_019790 did not produce a dark color in the supernatant when grown in liquid MM (data not shown).

AFUB_019790 encodes a putative transcription factor of 895 amino acids with a molecular weight of 99.8 kDa; a GAL4-like Zn(II)2Cys6 (or C6 zinc) binuclear cluster DNA-binding domain (SM00066; http://smart.embl-heidelberg.de/; between amino acids 63 to 107) and a fungal-specific transcription factor domain (SM000906; http://smart.embl-heidelberg.de/; between amino acids 416 to 489). AFUB_019790 has high sequence similarity at the protein level with Saccharomyces cerevisiae Asg1p (24% identity; 40% similarity; E value, 8e^−33^), a transcriptional regulator predicted to be involved in stress responses, as *ASG1* deletion strains have a respiratory deficiency, increased CFW sensitivity, and slightly increased cycloheximide resistance (19). Accordingly, we have named AFUB_019790 *asgA*. Phylogenetic analysis of AsgA across fungal species representing the 13 different taxonomic classes or subphyla within Dikarya (www.fungidb.org) revealed that orthologues were largely distributed in the *Pezizomycotina* (*Eurotiomycetes* [*Chaetothyriomycetes*], *Sordariomycetes*, *Leotiomycetes*, and *Dothideomycetes*), *Saccharomycotina* (*Saccharomycetes*), *Taphrinomycotina* (*Schizosaccharomycetes*), *Ustilaginomycotina* (*Ustilaginomycetes*), *Pucciniomycotina* (*Pucciniomycetes*), *Glomeromycotina* (*Glomeromycetes*), and *Chytridiomycota* (*Chytridiomycetes*) (Fig. 11C and Table S8 at https://doi.org/10.6084/m9.figshare.12869864). AsgA orthologues are also present in other important fungal pathogens, such as Botrytis cinerea, *Coccidioides* spp., *Sporothrix* spp., *Paracoccidioides* spp., *Fusarium* spp., and Ajellomyces capsulatus (Table S8 at https://doi.org/10.6084/m9.figshare.12869864).

We constructed the *asgA*-complemented strain and confirmed that deletion of this TF-encoding gene resulted in increased sensitivity to CR and CFW compared to the wild-type and complemented strains (Fig. 11D).

### The Δ*gprM* and Δ*gprJ* mutants have reduced virulence in the Galleria mellonella insect model of invasive aspergillosis.

**FIG 12:**
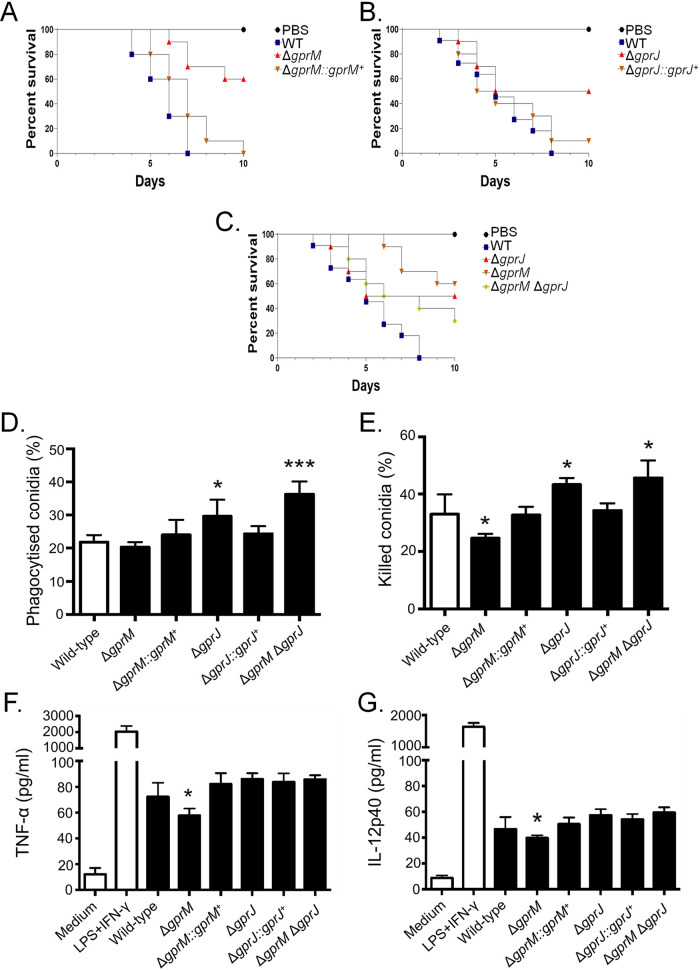
GprM and GprJ are important for A. fumigatus virulence in G. mellonella wax moth. (A to C) Survival curves of G. mellonella larvae (*n* = 10/fungal strain) infected via injection with 10^6^ conidia of the *gprM* and *gprJ* single- and double-deletion strains. Larval survival was monitored over a time period of 10 days. (D and E) Bone marrow-derived murine C57BL/6 macrophages (BMDMs) phagocytize (D) and kill (E) a higher number of Δ*gprJ* and Δ*gprM* Δ*gprJ* conidia *in vitro*, whereas they kill less Δ*gprM* conidia. (F and G) Concentrations of the tumor necrosis factor alpha (TNF-α) and interleukin 12 (IL-12) p40 cytokines in BMDMs infected with live-resting (LR) conidia, show that Δ*gprM* conidia elicit a reduced inflammatory response. Positive control, lipopolysaccharide plus interferon γ (LPS + IFN-γ). Standard deviations represent the average of three biological replicates, and all the strains were compared with the wild-type and complemented strains (*, *P* < 0.05 in a two-tailed, unpaired Student's *t* test).

We wondered whether GprM and GprJ are important for A. fumigatus virulence since they are involved in cell wall maintenance, which is crucial for pathogenicity (20). G. mellonella larvae (*n* = 10 for each strain) were infected with the WT, *gprM*, and *gprJ* deletion and complementation strains, and survival was assessed over a time period of 10 days (Fig. 12A to 12C). The wild‐type, Δ*gprM::gprM^+^*, and Δ*gprJ::gprJ^+^* strains caused 100, 100, and 90% mortality after 10 days postinfection (p.i.), respectively (Fig. 12A and B). In contrast, the Δ*gprM* and Δ*gprJ* strains caused 40 and 50% mortality rates 10 days p.i., respectively, which was statistically different (*P* < 0.001; Mantel-Cox and Gehan-Breslow-Wilcoxon tests) from the WT and complemented strains (Fig. 12A and B). The Δ*gprM* Δ*gprJ* strain caused 70% mortality at 10 days p.i., which was not statistically different from the WT and complemented strains (*P* < 0.001; Fig. 12C).

Since deletion of *gprM* and *gprJ* caused alterations in cell wall composition (Fig. 5), we hypothesized that this could influence the host immune response. Macrophages contribute to innate immunity, fungal clearance, and the generation of a proinflammatory response during A. fumigatus infection (21). The capacity of bone marrow-derived macrophages (BMDMs) to phagocytize and kill conidia derived from the WT, Δ*gprM*, Δ*gprM::gprM*^+^, Δ*gprJ*, Δ*gprJ::gprJ*^+^, and Δ*gprM* Δ*gprJ* strains was assessed. Conidia from the Δ*gprJ* and Δ*gprM* Δ*gprJ* strains are significantly more phagocytosed than conidia from the WT and Δ*gprJ::gprJ* strains, whereas no differences in phagocytosis were observed for the other strains (Fig. 12D). In agreement with the increased phagocytosis, conidia from the Δ*gprJ* and Δ*gprM* Δ*gprJ* strains were killed significantly more than the WT and gprJ-complemented strains (Fig. 12E). In addition, the Δ*gprM* conidia are killed less than conidia from the WT and Δ*gprM::gprM*^+^ strains (Fig. 12E). Interestingly, there is a reduced production of the cytokines tumor necrosis factor alpha (TNF-α) and interleukin 12 (IL-12) p40 in the Δ*gprM* strain compared to the WT and Δ*gprM::gprM*^+^ strains (Fig. 12F and G). There are no differences between these cytokines in the Δ*gprJ* and Δ*gprM* Δ*gprJ* strains compared to the WT and Δ*gprJ::gprJ^+^* strains (Fig. 12F and G).

Taken together, these results indicate that Δ*gprM* and Δ*gprJ* are important for A. fumigatus virulence in G. mellonella, whereas the double mutant is not, suggesting the existence of an unknown compensatory signaling mechanism that promotes virulence.

## DISCUSSION

Fungi can sense their environment through the presence of membrane-associated GPCRs, which activate downstream signaling cascades and allow the fungus to appropriately respond to environmental cues. In the case of human-pathogenic fungi, such as A. fumigatus, these membrane receptors are predicted to be crucial for mammalian host infection and are suggested targets for the development of antifungal drugs (6, 7, 22). In this work, we have characterized two A. fumigatus GPCRs and have shown their importance for several virulence determinants, such as the CWI pathway, secondary metabolite production, and infection.

To date, the genome of A. fumigatus is predicted to encode 15 GPCRs, which remain largely uncharacterized, with the exception of a couple of studies that functionally characterized the three GPCRs GprC, GprD, and GprK (10, 11). Furthermore, the great number of GPCRs in fungal genomes makes their study difficult because the majority of single-deletion mutants of GPCR genes do not show a clear phenotype due to the existence of redundancy between them (23–25). In agreement, deletion of *gprM* and *gprJ* resulted in two strains with increased melanin production in supernatants of liquid cultures. A function for GprM in repressing DHN-melanin production has previously been described (12). Furthermore, GprM physically interacts with the Gα protein GpaA, with deletion of *gpaA* resulting in a strain with increased DHN-melanin production (12). It was suggested that GprM-GpaA signaling occurs through the MAPK MpkB, resulting in the regulation of SM production, including the biosynthesis of DHN-melanin (12). Recently, this signaling cascade consisting of the three kinases SteC, MkkB, and MpkB, as well as the SteD adaptor protein and the HamE scaffold, was shown to be critical for the regulation of fungal development and secondary metabolism (26). In this study, we identified an additional GPCR, GprJ, that may also be involved in controlling DHN-melanin production. We are currently performing studies to determine whether GprJ also physically interacts with GpaA and whether it activates the MpkB signaling pathway. It is likely that GprJ at least participates in the regulation of the MpkB pathway, as GprJ was shown to regulate phosphorylation of MpkA, the MAPK of the CWI pathway which indirectly interacts with MpkB (12).

Furthermore, this study showed a genetic interaction between *gprM* and *gprJ*, which appears to be highly complex. In the Δ*gprM* and Δ*gprJ* strains, similar phenotypes are observed for cell wall polysaccharide concentrations and virulence in G. mellonella. Simultaneous deletion of both GPCR-encoding genes resulted in a strain with a cell wall and virulence similar to the WT strain, but not the MpkA phosphorylation profile, suggesting that the absence of both receptors activates unknown compensatory signaling mechanisms to promote normal MpkA-independent cell wall organization and virulence. Transcriptional profiling of both single-deletion strains showed that few genes are targeted by both GPCRs, suggesting that GprM- and GprJ-mediated signaling differs significantly and may occur via different pathways. This is supported by the observation that PKA activity is decreased in the Δ*gprM* and Δ*gprM* Δ*gprJ* strains but not in the Δ*gprJ* strain. Restauration of WT phenotypes upon deletion of two genes encoding GPCRs has already been observed in other fungi. In A. nidulans, the addition of glucose after carbon starvation resulted in a burst in cAMP, a response that was absent in the Δ*gprH*, Δ*gprI*, and Δ*gprM* single-deletion strains (17). In contrast, the simultaneous absence of multiple GPCRs in the double- or triple-deletion strains resulted in the recovery of the cAMP burst after addition of glucose (17). These studies show that phenotypes observed in single-deletion strains can cancel each other out and that this may also be the case for the Δ*gprM* Δ*gprJ* strain for certain conditions, as these GPCRs may regulate signaling pathways in an opposite manner.

It is clear, though, from our study that GprM and GprJ, as well as downstream signaling events, are involved in SM production in A. fumigatus. This is in agreement with previous studies which showed that the deletion of the G-protein α subunit, GpaB, and the adenylate cyclase AcyA decreased DHN-melanin production in A. fumigatus (27), while overexpression of the protein kinase A catalytic subunit PkaC1 induced the expression of the DHN-melanin cluster genes (28). Genes encoding proteins required for the biosynthesis of SMs were shown to be under the transcriptional control of GprM and GprJ. Indeed, GprM and GprJ are involved in the repression of SM secretion, as the concentrations of secreted SMs such as fumagillin, pyripyropene, fumigaclavine C, fumiquinazoline, melanin, and fumitremorgin were significantly reduced in the Δ*gprM* and Δ*gprJ* strains. This is in agreement with a study that showed that GprM negatively regulates DHN-melanin production (12). GprM- and GprJ-mediated regulation of SM biosynthesis may occur via different MAPK signaling pathways, including MpkB (as discussed above) and MpkA. Extracellular concentrations of pyripyropene, fumigaclavine C, fumiquinazoline, and melanin were not detected in the *mpkA*-null mutant, while fumagillin and fumitremorgin are negatively regulated by MpkA, suggesting that regulation through MpkA occurs in a metabolite-dependent manner. MpkA was indeed shown to be important for the regulation of genes encoding proteins required for gliotoxin, pyomelanin, pseurotin A, and siderophore biosynthesis (29). The Δ*mpkA* strain showed reduced DHN-melanin and gliotoxin production but had increased production of siderophores during iron starvation conditions (16, 29–31). Regulation of SM production is therefore likely to occur via various signaling pathways that are activated upon sensing many different environmental cues. Adding to this complexity is the existence of cross talk between different GPCR downstream signaling pathways, which has been shown to regulate a number of virulence determinants, including SM production and CWI, in A. fumigatus (12, 18). In the plant pathogen A. flavus, deletion of the 15 GPCR-encoding genes resulted in strains with one or more defects in growth, development, aflatoxin production, response to carbon sources, nitrogen sources, stress agents, and lipids, suggesting cross talk between downstream signaling pathways (23). It is tempting to suggest that each extracellular signal results in the activation of a common and “core” signaling pathway which interacts with a specific set of proteins to fine-tune the cellular response to the environmental cue.

Furthermore, GprM and GprJ were shown to regulate A. fumigatus CWI, a process that is likely to occur through the MAPK MpkA. Phosphorylation of MpkA is synonymous with CWI pathway activation (16). Deletion of *gprM* and *gprJ* resulted in an accumulation of phosphorylated MpkA and thus a “hyperactive” CWI pathway. This activation of CWI signaling may be the cause for the observed accumulation of cell wall polysaccharides and significantly increased cell wall thickness. In contrast, overexpression of *gprM* and *gprJ* reduced MpkA phosphorylation and inactivated the CWI pathway. As a consequence, cell wall thickness was significantly reduced, and strains were sensitive to cell wall-interfering agents. Although the CWI pathway is well characterized in A. fumigatus, less is known about MpkA downstream targets. A notable exception is the TF RlmA, which acts downstream of MpkA and is involved in MpkA phosphorylation and required for a normal cell wall organization and for vegetative growth (32). Recently, the TF ZipD, together with the calcium-dependent TF CrzA, was shown to also influence MpkA phosphorylation levels and to be important for cell wall maintenance through regulating the expression of glucan and chitin biosynthetic genes in a calcium-dependent manner (33, 34). To identify additional TFs that act downstream of the GprM-MpkA signaling pathway, we selected 44 TF deletion strains whose expression was significantly altered in our *gprM* overexpression RNA-seq data and assessed their growth in the presence of cell wall-interfering agents. The Δ*asgA* was significantly more sensitive to cell wall-perturbing agents but did not produce a dark pigment in the supernatants when grown in liquid medium, suggesting it is not involved in the repression of the melanin production. In agreement with its role in cell wall maintenance, AgsA was shown to have increased phosphorylation upon CR exposure (35). We are currently performing additional work to decipher the relationship between AgsA and MpkA in the presence of cell wall stress.

In summary, this study functionally describes two A. fumigatus GPCRs and significantly contributes to our understanding of these types of signaling modules in this opportunistic fungal pathogen. Furthermore, this study also shows the complexity and cross talk that underlie fungal GPCR signaling in regulating important virulence traits. As fungal GPCRs are poorly conserved in animals and plants and regulate virulence determinants in pathogenic fungi, they continue to be interesting targets for the development of novel antifungal drug and combinational therapies.

## MATERIALS AND METHODS

### GPCRs among *Aspergillus* species.

To identify GPCRs across *Aspergillus* species, we used a best BLAST hit-based approach (36). More specifically, we first obtained *Aspergillus* GPCR sequences based on a previously published list (9). Using the *Aspergillus* GPCR protein sequences, we used BLAST+ v2.3.0 (36) to identify GPCRs among 52 publicly available and annotated *Aspergillus* genomes from NCBI’s GenBank database (download date, 22 June 2018). GPCR sequences were aligned and trimmed as described elsewhere (37). Briefly, sequences were aligned using MAFFT v7.402 (38) (parameters, --bl 62, --op 1.0, --maxiterate 1000, --retree 1, --genafpair). Nucleotide sequences were then threaded onto the protein alignment using Biopython v1.7 (39) and trimmed using trimAl with the gappyout parameter (40). The resulting trimmed alignment was used to infer the evolutionary relationships among GPCRs using IQ-TREE v1.6.1 (41). Bipartition support was assessed using 5,000 ultrafast bootstrap approximations (42). Bipartitions with less than 85% ultrafast bootstrap approximation support were collapsed.

### Strains and media.

Strains were grown at 37°C in either complete medium (YAG; 2% [wt/vol] glucose, 0.5% [wt/vol] yeast extract, trace elements) or minimal medium (MM; 1% [wt/vol] glucose, nitrate salts, trace elements, pH 6.5). Solid YAG and MM were the same as described above except that 1.7% (wt/vol) or 2% (wt/vol) agar was added. Trace elements, vitamins, and nitrate salts compositions were as described previously (43).

For phenotypic characterization, solid medium plates were inoculated with 10^4^ conidia per strain and left to grow for 120 h at 37°C. Radial growth was expressed by the ratio of colony radial diameter in the stress condition by colony radial diameter in the control (no stress) condition. All the strains used in this work are described in Table S1 at https://doi.org/10.6084/m9.figshare.12869864.

### Enzymatic assays.

PKA activity (PepTag non-radioactive protein kinase assay kit V5340; Promega) was carried out according to the manufacturer’s instructions. Extracellular glucose concentrations were quantified as described previously (17).

### Aspergillus fumigatus strains.

Fungi strains and plasmids used in this study are listed in Table S1 at https://doi.org/10.6084/m9.figshare.12869864. In addition, all primers used in this work are listed in Table S9 at https://doi.org/10.6084/m9.figshare.12869864.

The *gprJ* (Afu1g06840) gene was deleted by gene replacement with the *ptrA* gene from A. oryzae using homologous recombination (44) generating the Δ*gprJ* strain. Briefly, the 5′ and 3′ flanking regions of the *gprJ* gene were amplified from A. fumigatus genomic DNA (gDNA) using specific primers (P1 and P2 and P3 and P4, respectively) (Table S9 at https://doi.org/10.6084/m9.figshare.12869864). These were subsequently fused by using S. cerevisiae *in vivo* recombination (45) to the *ptrA* gene, which confers resistance to pyrithiamine and which was PCR amplified from plasmid pSK275 (primers P5 and P6). The construct was used to transform the A. fumigatus Δ*akuB*^KU80^ wild-type strain via homologous recombination (46). Mutants were selected and purified on MM containing 1 μg ml^−1^ pyrithiamine and confirmed with PCR using a primer (P29) annealing externally to the integrating cassette (Fig. S3 at https://doi.org/10.6084/m9.figshare.12869864).

The Δ*gprJ* strain was cultivated on complete medium supplemented with uridine, uracil, and 0.75 mg ml^−1^ of 5-fluoroorotic acid to obtain a Δ*gprJ pyrG^−^* strain. The Δ*gprJ* pyrG- strain was confirmed by PCR and used to generate the *gprJ*-complementing strain, the Δ*gprJ* Δ*gprM* and Δ*gprJ* Δ*pksP* double mutants. Deletion cassettes containing the *pyrG* selectable marker were generated via yeast-mediated recombination as previously described (47). Briefly, 1-kb fragments from the gene 5′ and 3′ flanking regions were amplified with the corresponding primer pairs (primers P13 through P16 for the *gprM* gene [AspGD database no. Afu7g05300] and P31 through P34 for *pksP* [AspGD database no. Afu2g17600]). The *pyrG* gene fragment was PCR amplified from the plasmid pCDA21 with primers P11 and P12. All fragments were cotransformed into the Saccharomyces cerevisiae SC9721 strain along with the linearized plasmid pRS426. Deletion cassettes were finally amplified from yeast genomic DNA with corresponding 5′ forward and 3′ reverse primers and used to transform the auxotroph Δ*gprJ pyrG^−^* strain. Mutants were selected and purified in MM and were checked by PCR using external primers (P29 for *gprM* and P35 for *pksP*). To complement the Δ*gprJ* strain, a complementation cassette containing the *gprJ* ORF as well as the *pyrG* selectable marker was constructed as described above and used to transform the Δ*gprJ pyrG^−^* strain. Primers P8 and P9, containing homologous tags to the *pyrG* gene, were used to generate the cassette. The complementing cassette was transformed into the Δ*gprJ pyrG^−^* strain, and positive A. fumigatus-complementing candidates were selected, purified through three rounds of growth on plates, submitted to gDNA extraction, and confirmed by PCR (Fig. S3 at https://doi.org/10.6084/m9.figshare.12869864).

The overexpressing *xylp::gprJ* strain containing the *gprJ* gene N-terminally tagged to the xylose reductase promoter (*xylp*) and C-terminally tagged to hemagglutinin (HA) plus the TRPC terminator and the *pyrG* auxotrophic marker was generated by homologous integration. The final cassette contained the following fragments: 5′ untranslated region (UTR) (P7/P23), *xylp* (P17/P18), *gprJ* open reading frame (ORF) without the stop codon (P21/P22), 3xHA-*trpC*-*pyrG* (P19/P20), and 3′ UTR (P24/P25). Primer pairs used for PCR amplification of each DNA fragment are indicated in parentheses. Fragments *xylp* and 3xHA-*trpC*-*pyrG* were PCR amplified from the plasmids pYES-pXyl-hph-devR and pOB430, respectively. All fragments were cotransformed into S. cerevisiae SC9721, and the resulting cassette was used to transform the Δ*akuB*^KU80^ *pyrG^−^* strain. Genomic integration of the cassette was verified by PCR (Fig. S3 at https://doi.org/10.6084/m9.figshare.12869864), and the functionality of the construction was assayed by real-time PCR (primers P26 and P27) (Fig. S3 at https://doi.org/10.6084/m9.figshare.12869864).

Complementation of the *asgA* gene (Afu2g02690) was achieved via cotransformation. A fragment containing the *asgA* gene flanked by 1-kb 5′ and 3′ UTR regions was PCR amplified (primers P35 and P36). This DNA fragment was transformed into the Δ*asgA* strain along with the plasmid pPTR1 (TaKaRa Bio) containing the pyrithiamine resistance *ptrA* gene. Mutants were selected on MM supplemented with 1 μg ml^−1^ pyrithiamine and confirmed with PCR using the external primer (P37).

### Generation, validation, and screening of transcription factor knockout mutants.

The transcription factor knockout mutant collection was generated in the A. fumigatus strain A1160 (a derivative of CEA17, ΔKu80 pyrG^+^) according to reference 48. The most sensitive and resistant mutants to CR (Congo red) and CFW (calcofluor white) were identified by growing them on MM plus CR and MM plus CFW for 5 days at 37°C; they were retested and purified for further characterization.

### Transcriptome sequencing, cDNA synthesis, and real-time PCR.

For transcriptome sequencing, A. fumigatus conidia (5 × 10^7^) from the CEA17, Δ*gprM*, and Δ*gprJ* strains were inoculated in triplicate in liquid MM and cultured for 24 h at 37 °C. For the *xylP::gprM* and *xylp::gprJ* strains, the conidia were inoculated in MM plus 1% glucose, grown for 24 h at 37°C, and then transferred to MM plus 1% xylose for 4 or 1 h, respectively. Mycelia were harvested, frozen, and ground in liquid nitrogen. Total RNA was extracted using TRIzol (Invitrogen), treated with RQ1 RNase-free DNase I (Promega), and purified using the RNAeasy kit (Qiagen) according to the manufacturer's instructions. RNA from each treatment was quantified using a Qubit fluorometer and analyzed using an Agilent 2100 Bioanalyzer system to assess the integrity of the RNA. All RNA had an RNA integrity number (RIN) between 7.0 and 9.5.

The Illumina TruSeq Stranded mRNA sample preparation kit was used to construct cDNA libraries following the manufacturer's instructions. Libraries were sequenced (2× 100 bp) on the Brazilian Bioethanol Science and Technology Laboratory's (CTBE) next-generation sequencing (NGS) facility using a HiSeq 2500 instrument, generating approximately 11 × 10^6^ fragments per sample.

Obtained fastq files were quality checked with FastQC (http://www.bioinformatics.babraham.ac.uk/projects/fastqc/) and cleaned (quality trim, adaptor removal, and minimum length filtering) with Trimmomatic (49). rRNA was removed using SortMeRNA (50). High-quality RNA-seq reads were mapped against the A. fumigatus genome using the CLC Genomics Workbench software (CLC bio v4.0; Finlandsgade, Denmark) using the following parameters: mapping settings (minimum length fraction, 0.7; minimum similarity fraction, 0.8; maximum number of hits for read, 1) and alignment settings (minimum distance, 180; maximum distance, 1,000). All samples achieved saturation of known exon-exon junctions. Reproducibility among biological replicates was assessed by exploring a principal-component analysis (PCA) plot of the top 500 genes that have the largest biological variation between the libraries and by pairwise measuring the Pearson correlation among the replicates over the whole set of genes. In order to assess transcript abundance, exonic reads were counted in a strand-specific way using the featureCounts function from Rsubread Bioconductor package (51). Calling of differentially expressed genes was carried out using DESeq2 (58) using a threshold-adjusted *P* value of <0.01 (52).

For quantitative real-time PCR analysis, 1 × 10^7^ spores of the wild-type, *xylP::gprM*, and *xylp::gprJ* strains were inoculated in 50 ml of liquid MM supplemented with 1% glucose and grown at 37°C. After 24 h of growth, mycelia were transferred to liquid MM supplemented with 1% xylose for up to 4 h. Then, mycelia were frozen in liquid nitrogen, and the total cellular RNA was extracted using TRIzol (Invitrogen) according to the manufacturer’s instructions. Total RNA was purified (RNeasy minikit; Qiagen), according to the manufacturer’s instructions, and the quality of the RNA was assayed using the Agilent Bioanalyzer 2100 (Agilent Technologies). RNA was reverse transcribed to cDNA using the ImProm-II reverse transcription system (Promega), and the synthesized cDNA was used for real-time analysis using the SYBR green PCR master mix kit (Applied Biosystems) in the ABI 7500 Fast real-time PCR system (Applied Biosystems, Foster City, CA, USA). Primer sequences are listed in Table S9 at https://doi.org/10.6084/m9.figshare.12869864.

### Immunoblot analysis.

To assess the phosphorylation status of MpkA, fresh harvested conidia (1 × 10^7^) of the wild-type and deletion strains were inoculated in 50 ml liquid MM at 37°C for 24 h (180 rpm). For the overexpression strains, the wild-type and the mutant strains were grown at 37°C for 24 h in MM and transferred for MM plus 1% xylose at 37°C for 30 to 240 min. Mycelia were ground into liquid nitrogen with a pestle and mortar. For protein extraction, 0.5 ml lysis buffer containing 10% (vol/vol) glycerol, 50 mM Tris-HCl pH 7.5, 1% (vol/vol) Triton X-100, 150 mM NaCl, 0.1% (wt/vol) SDS, 5 mM EDTA, 50 mM sodium fluoride, 5 mM sodium pyrophosphate, 50 mM β-glycerophosphate, 5 mM sodium orthovanadate, 1 mM phenylmethylsulfonyl fluoride (PMSF), and 1× complete mini protease inhibitor (Roche Applied Science) was added to the ground mycelium. Extracts were centrifuged at 20,000 × *g* for 40 min at 4°C. The supernatants were collected, and the protein concentrations were determined using the Bradford assay (Bio-Rad). Fifty micrograms of protein from each sample were resolved in 12% (wt/vol) SDS-PAGE and transferred to polyvinylidene difluoride (PVDF) membranes (Merck Millipore). The phosphorylated fractions of the MAP kinase, MpkA, were examined using anti-phospho-p44/42 MAPK antibody (Cell Signaling Technologies) following the manufacturer’s instructions using a 1:1,000 dilution in TBST buffer (137 mM NaCl, 20 mM Tris, and 0.1% Tween 20). Anti-γ-actin was used to normalize the protein loading. The primary antibody was detected using a horseradish peroxidase (HRP)-conjugated secondary antibody raised in rabbit (Sigma). Chemiluminescent detection was achieved using an ECL Prime Western blotting detection kit (GE Healthcare). To detect these signals on blotted membranes, the ECL Prime Western blotting detection system (GE Healthcare, Little Chalfont, UK) and LAS1000 (Fujifilm, Tokyo, Japan) were used. The images generated were subjected to densitometric analysis using ImageJ software (http://rsbweb.nih.gov/ij/index.html).

### Secondary metabolite extraction and high-resolution mass spectrometry analyses.

For mass spectrometry analyses, extractions were performed according to reference 53 with modifications. Briefly, 100 mg of the fungal lyophilized supernatant was extracted with 1 ml of MeOH during 40 min in ultrasonic bath. The extracts were centrifuged at 13,000 rpm for 1 min, and the supernatants were collected and dried under N_2_. Crude extracts were resuspended in 600 μl MeOH and the extracted secondary metabolites were filtered through a 0.22 μM pore size filter. All the extractions were performed in triplicate.

The liquid chromatography-high-resolution mass spectrometry (LC-HRMS) analyses were performed in a liquid chromatography-mass spectrometer LC Agilent 1200 coupled to an Agilent iFunnel 6550 quadrupole time of flight mass spectrometry (Q-ToF) LC-MS with an electrospray ionization (ESI) source. All the operation and spectra analyses were conducted using Agilent MassHunter Workstation software. The parameters of MS analyses were set as follows: ESI source in positive mode, nebulizing gas temperature at 290°C, capillary voltage at +3,000 V, nozzle voltage at 320 V, drying gas flow of 12 ml min^−1^, nebulization gas pressure of 45 lb/in^2^, auxiliary gas temperature at 350°C, auxiliary gas flow of 12 ml min^−1^, and mass range of *m/z* 100 to 1700. The chromatographic separation was performed on a Thermo Scientific Accucore C_18_ column (2.6 μm, 2.1 mm by 100 mm), and 0.1% formic acid (A) and acetonitrile (B) were used as mobile phase. The eluent profile (A:B) was 0 to 10 min, gradient from 95:5 to 2:98; 10 to 15 min and final isocratic elution with 2:98. The flow rate was set at 0.2 ml min^−1^, and 1 μl of sample was injected in each injection.

The low-resolution mass spectrometry analyses were performed in an Acquity ultraperformance LC system coupled to a Quattro Micro triple quadrupole mass spectrometer from Waters. The same chromatographic conditions were applied as before. The mass spectrometer parameters were as follows: capillary voltage, 3.0 kV; cone voltage, 20 V; extractor voltage, 3 V; source temperature, 150°C; desolvation temperature, 350°C; and desolvation gas flow, 600 liters/h. Dry nitrogen was used as desolvation and nebulization gas, and argon was used as collision gas. All the data were acquired and processed using MassLynx v4.1 software (Waters).

### Cell wall polysaccharides extraction and sugar quantification.

Fungal cell wall polysaccharides were extracted from 10 mg dry-frozen biomass as described previously (54). One milliliter of extracted samples was concentrated 10× by lyophilization, and sugars were subsequently analyzed by high-performance liquid chromatography (HPLC) using a Young Lin YL9100 series system (Young Lin, Anyang, South Korea) equipped with a YL9170 series refractive index (RI) detector at 40°C. Samples were loaded in a Rezex ROA (Phenomenex, USA) column (300 by 7.8 mm) at 85°C and eluted with 0.05 M sulfuric acid at a flow rate of 1.5 ml/min.

### Transmission electron microscopy analysis of cell wall.

Strains were grown statically from 1 × 10^7^ conidia at 37°C in MM for 24 h. Mycelia were harvested and immediately fixed in 0.1 M sodium phosphate buffer (pH 7.4) containing 2.5% (vol/vol) of glutaraldehyde and 2% (wt/vol) of paraformaldehyde for 24 h at 4°C. Samples were encapsulated in agar (2% wt/vol) and subjected to fixation (1% OsO_4_), contrasting (1% uranyl acetate), ethanol dehydration, and a two-step infiltration process with Spurr resin (Electron Microscopy Sciences) of 16 h and 3 h at room temperature (RT). Additional infiltration was provided under vacuum at RT before embedment in BEEM capsules (Electron Microscopy Sciences) and polymerization at 60°C for 72 h. Semithin (0.5-μm) survey sections were stained with toluidine blue to identify the areas of best cell density. Ultrathin sections (60 nm) were prepared and stained again with uranyl acetate (1%) and lead citrate (2%). Transmission electron microscopy (TEM) images were obtained using a Philips CM-120 electron microscope at an acceleration voltage of 120 kV using a MegaView3 camera and iTEM 5.0 software (Olympus Soft Imaging Solutions GmbH). Cell wall thickness of 100 sections of different germlings was measured at ×23,500 magnification and images analyzed with the ImageJ software (55). Statistical differences were evaluated by using one-way analysis of variance (ANOVA) and Tukey’s *post hoc* test.

### Preparation of BMDMs and cytokine quantification.

BMDMs were prepared and isolated as described previously (34, 56). Bone marrow cells were obtained from femur and tibia of adult (6 to 8 weeks of age) male C57BL/6 mice. The BMDMs were plated on 48-well plates (1.5 × 10^6^ cells/ml; 7.5 × 10^5^ cells/well), and the cultures were maintained for 48 h for quantification of cytokines. The cytokine measurement was quantified using an enzyme-linked immunosorbent assay (ELISA) kit, according to the protocol of the manufacturer (BD Biosciences, Pharmingen, San Diego, CA, USA).

### Phagocytosis and killing assay.

BMDMs were used in all phagocytosis and killing assays as described previously (34, 56). The phagocytic index was obtained using 2 × 10^5^ cells/well plated on glass 13-mm-diameter coverslips placed on 24-well plates. BMDMs were treated with the conidia of fungi (2 × 10^5^ conidia; macrophages/conidia, 1:1) for 4 h at 37°C with 5% CO_2_. An average of 100 macrophages was enumerated to determine the percentage of conidia that were ingested per macrophage. For the killing assay, the BMDMs were treated with 5 × 10^5^ conidia (macrophages/conidia, 1:1) for 48 h at 37°C with 5% CO_2_. Serial dilutions were prepared using the lysate, and the cells were then plated and incubated at 37°C for 48 h. The viable fungi were enumerated, and the CFU counts per milliliter were calculated. The experiments were repeated three times, each performed in triplicate.

### Virulence analysis in Galleria mellonella models.

The Galleria mellonella larvae were obtained by breeding adult larvae (57) weighing 275 to 330 mg kept starving in petri dishes at 37° in the dark for 24 h prior to infection. All selected larvae were in the final stage of larval (sixth) stage development. Fresh conidia of each strain of A. fumigatus were counted using a hemocytometer, and the initial concentration of the conidia suspensions for the infections were 2 × 10^8^ conidia/ml. A total of 5 μl (1 × 10^6^ conidia/larvae) of each suspension was inoculated per larva. The control group was composed of larvae inoculated with 5 μl of phosphate-buffered saline (PBS) to observe death by physical trauma. The inoculum was performed using the Hamilton syringe (model 7000.5 KH) through the last left prolegate. After infection, the larvae were kept at 37°C in petri dishes in the dark and scored daily. Larvae were considered dead due to lack of movement in response to touch. The viability of the inoculum administered was determined by plating a serial dilution of the conidia on YAG medium and incubating the plates at 37°C for 72 h.

### Data availability.

The RNA-seq data set can be accessed at NCBI’s Short Read Archive under the BioProject accession no. PRJNA634102.
